# Supplementary material for: Fucoidan from Lessonia trabeculata Induces Apoptosis through Caspase Dependent and Caspase-Independent Activation in 4T1 Breast Adenocarcinoma In Vitro
Source: Mar Drugs. 2024 May 29;22(6):251. doi: 10.3390/md22060251 (PMC11205089; doi:10.3390/md22060251)
Supplement: Supplementary file 1 [file marinedrugs-22-00251-s001.zip › marinedrugs-2941085-supplementary.pdf]

Supplementary figure

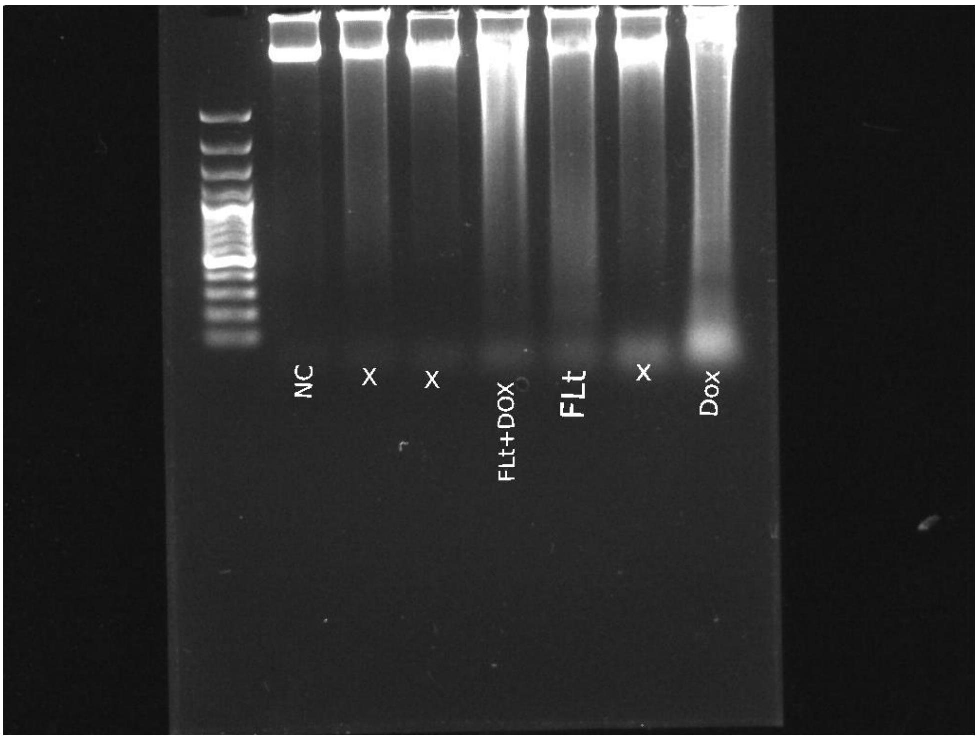

Fig. S1. Original gel image corresponding to Fig. 3.

The products were detected by 1.7 % agarose gel electrophoresis.

Results irrelevant to this study were marked with “X”.

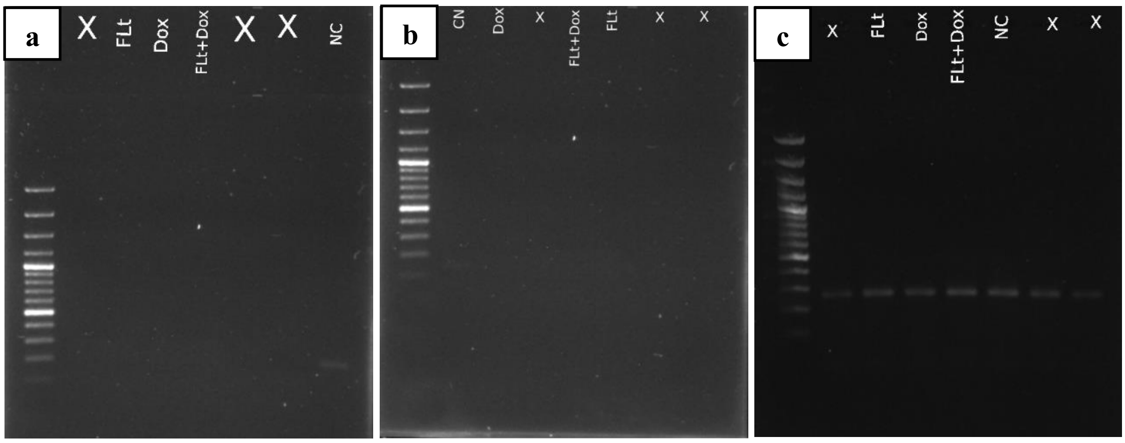

Fig. S2. Original gel images (a,b,c) corresponding to Fig. 4A.
The products were detected by 1.5 % agarose gel electrophoresis.
Results of the electrophoresis run (in duplicate) of the Bcl-2 gene. β-actin gene (c) was used as an internal control and original gel image was also shown.

Results irrelevant to this study were marked with “X”.

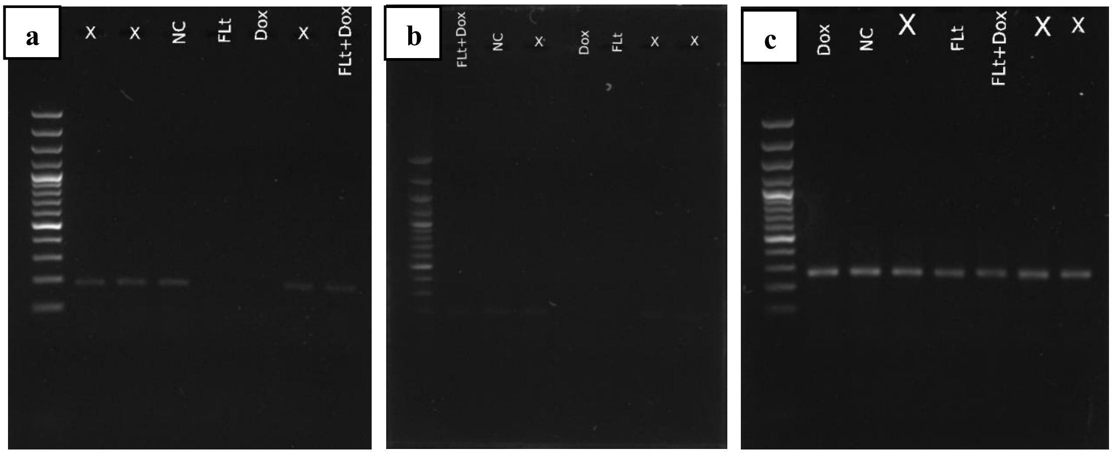

Fig. S3. Original gel images (a,b,c) corresponding to Fig. 4A.
The products were detected by 1.5 % agarose gel electrophoresis.
Results of the electrophoresis run (in duplicate) of the XIAP gene. β-actin gene (c) was used as an internal control and original gel image was also shown.

Results irrelevant to this study were marked with “X”.

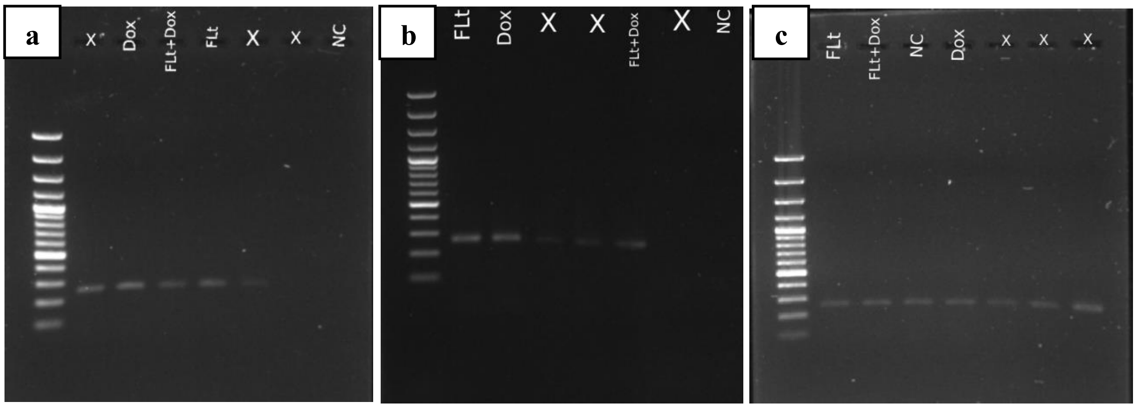

Fig. S4. Original gel images (a,b,c) corresponding to Fig. 4C.

The products were detected by 1.5 % agarose gel electrophoresis.
Results irrelevant to this study were marked with “X”. β-actin gene (c) was used as an internal control and original gel image was also shown.

Results of the electrophoresis run (in duplicate) of the Casp8 gene.

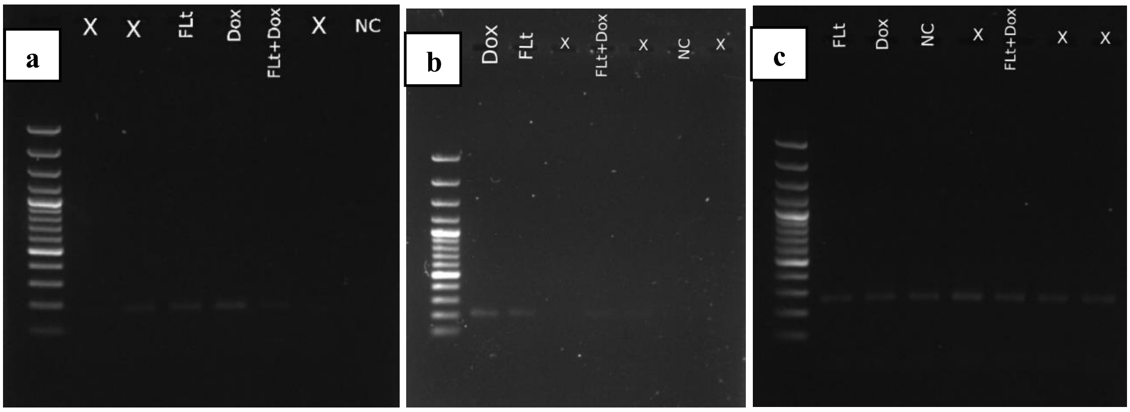

Fig. S5. Original gel images (a,b,c) corresponding to Fig. 4C.
The products were detected by 1.5 % agarose gel electrophoresis.
Results irrelevant to this study were marked with “X”. β-actin gene (c) was used as an internal control and original gel image was also shown.

Results of the electrophoresis run (in duplicate) of the Casp9 gene.

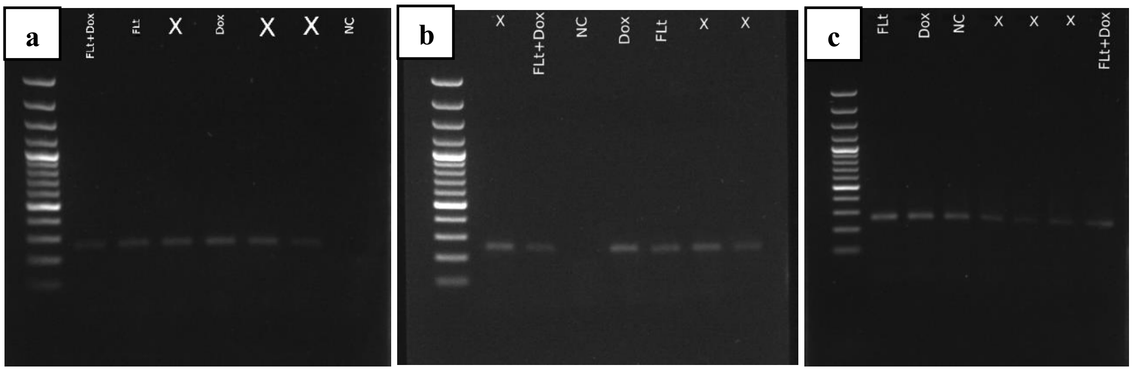

Fig. S6. Original gel images (a,b,c) corresponding to Fig. 4C.
The products were detected by 1.5 % agarose gel electrophoresis.
Results irrelevant to this study were marked with “X”. β-actin gene (c) was used as an internal control and original gel image was also shown.

Results of the electrophoresis run (in duplicate) of the AIF gene.
